# Ophiopogonin D: review of pharmacological activity

**DOI:** 10.3389/fphar.2024.1401627

**Published:** 2024-07-18

**Authors:** Ke-qian Chen, Shu-zhi Wang, Hai-bo Lei, Xiang Liu

**Affiliations:** ^1^ Department of Clinical Pharmacy, Xiangtan Central Hospital, Xiangtan, China; ^2^ Institute of Pharmacy and Pharmacology, School of Pharmaceutical Sciences, Hengyang Medical School, University of South China, Hengyang, China

**Keywords:** *Ophiopogon japonicus*, Ophiopogon D, pharmacology, pharmacokinetics, drug

## Abstract

**Background:**

Ophiopogon D is an important natural organic compound in *Ophiopogon japonicus*, which often has significant biological activity.

**Purpose:**

The purpose of this review is to systemically summarize and discuss the pharmacological activity and underlying mechanisms of OP-D in recent years.

**Method:**

PubMed and Web of Science were searched with the keywords:“*Ophiopogon japonicus*”, “Ophiopogon D” “pharmacology”, and “pharmacokinetics”. There was no restriction on the publication year, and the last search was conducted on 1 Jan 2024.

**Results:**

Emerging evidence suggests that OP-D possess numerous pharmacological activities, including bone protection, cardiovascular protection, immune regulation, anti-cancer, anti-atherosclerosis, anti-inflammatory and anti-NAFLD.

**Conclusion:**

OP-D has a potential value in the prevention and treatment of many diseases. We hope that this review will contribute to therapeutic development and future studies of OP-D.

## 1 Introduction

**FIGURE 1:**
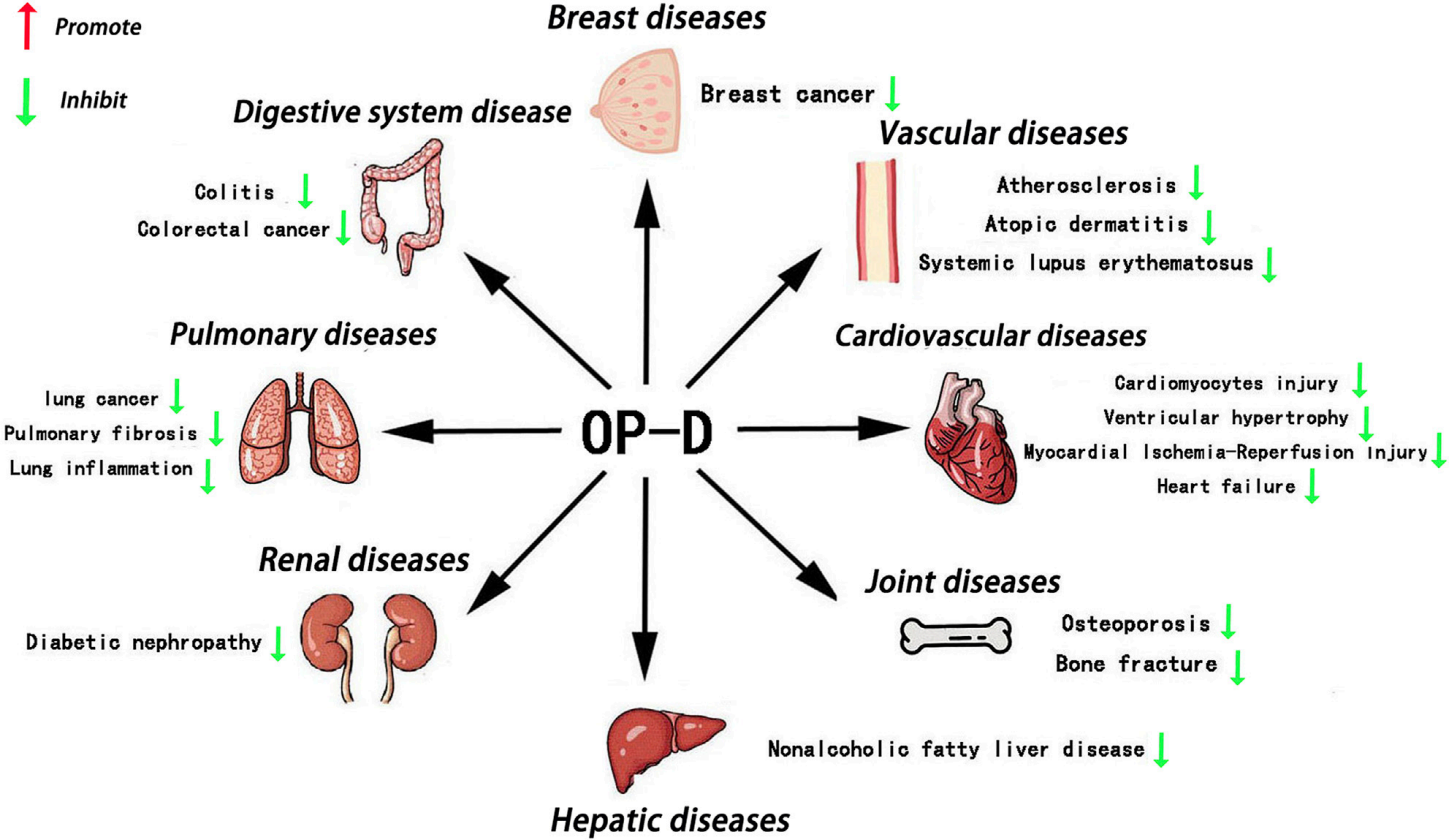
Main roles of OP-D in various tissues OP-D plays an important role in various organs, such as the bone, blood vessel, heart, breast, kidney, liver, lungs, colon and so on.

Since the 21st century, researchers have increasingly turned their attention to traditional Chinese herbs, recognizing their advantage of reduced side effects (Zeng et al., 2019). *Ophiopogon japonicus*, a well-known Chinese herb, has long been considered a health-promoting substance (Fang et al., 2018). At the same time, *Ophiopogon japonicus* is also a popular ornamental plant in East Asia (Yuan et al., 2019). Literature investigation has shown that *Ophiopogon japonicus* contains many active compounds, such as Dwarf Lilyturf Tuber-13 (DT-13), Ophiopogon-B(OP-B), Ophiopogon-D (OP-D), Liriopesides-B (LP-B), Ruscogenin (RUS), and Ophiopogon-D′ (OP-D′) (Ren-ping et al., 2014; Dong et al., 2021; Liu et al., 2023). OP-D is a rare C27 steroid glycoside isolated from the tuber of *Ophiopogon japonicus*. Over the past few years, extensive research on OP-D across animal and human models has demonstrated its multifaceted benefits, including anti-inflammatory, anti-cancer, anti-atherosclerosis, anti-NAFLD, immunomodulatory, osteoprotective, and cardioprotective effects (Figure 1). However, there is still a lack of comprehensive and critical review of OP-D pharmacological activity. We hope that this review will contribute to therapeutic development and future studies of OP-D.

## 2 Chemical properties and plant sources of OP-D

**FIGURE 2:**
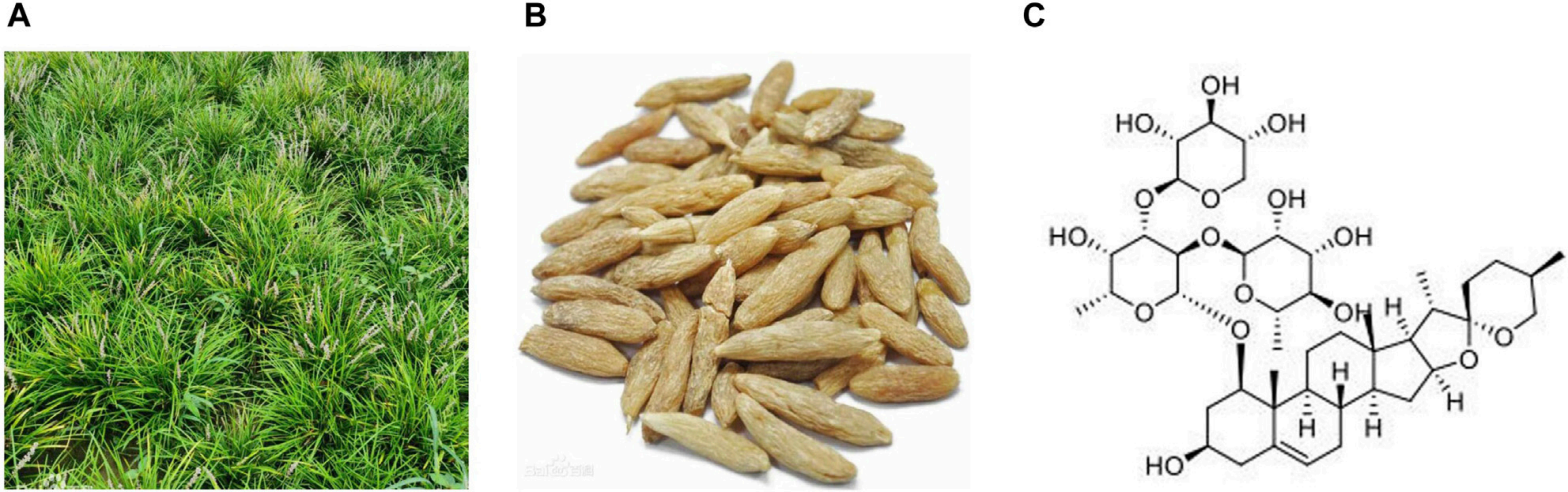
The whole plant **(A)**, roots **(B)** and structures **(C)** of OP-D.

*Ophiopogon japonicus* is a perennial bushy herb characterized by its bushy growth and small, oval or spindle-shaped roots, typically found in the middle or near the ends of the root system. The small tuberous roots are light brownish-yellow and very short (Figure 2). The leaf base is clumped. The seeds are spherical, and the flowers are solitary or in pairs. The flowering period is from May to August, and the fruiting period lasts from August to September (Lei et al., 2021). OP-D is identified as a white crystalline powder with a molecular formula of C_44_H_70_O_16,_ and a molecular weight of 855.07. It is soluble in methanol, ethanol, and dimethyl sulfoxide (DMSO), and features eight OH groups and six CH3 groups (Chen et al., 2016) (Figure 2).

## 3 Pharmacokinetics

Liquid chromatography (LC) and electrospray ionization mass spectrometry (ESI-MS) are two high-performance physical separation techniques, particularly for the detection of OP-D and its pharmacokinetics in rats. These two methods exhibited a good correlation coefficient over the investigated concentration range (r > 0.997, LLOD < 2.0 ng/mL, RSD < 7.5% and accuracies were 97.5%–107.0%) (Xia C. et al., 2008). The two-compartment pharmacokinetic model describes the evolution of drug levels in the organism by depicting the body as two pharmacokinetic compartments (the central and the peripheral compartments, also commonly referred to as compartment 1 and compartment 2, in that order). After intravenous dosing (77.0 μg/kg), the plasma concentration-time profile for OP-D was best fitted to an open two-compartment model (Cl = 0.024 ± 0.010 L/min/kg, T_1/2_ = 17.29 ± 1.70 min). As a component of ‘SHENMAI’ injection, the pharmacokinetics of OP-D revealed a significantly smaller clearance compared with a pure OP-D compound (Xia CH. et al., 2008). OP-D also influences on the pharmacokinetics and transport of other drugs. As a natural quinone compound, cryptotanshinone was found to have anti-inflammatory activities, anti-cancer activities, anti-metabolic disorders, cardiovascular protection and other functions. OP-D significantly increased the C_max_ and T_1/2_ of cryptotanshinone, while decreasing the clearance rate of cryptotanshinone. *In vitro*, OP-D improved metabolic stability by lowering intrinsic clearance and dramatically inhibited the transport of cryptotanshinone through a reduced efflux rate. The combination of cryptotanshinone and OP-D could inhibit the transport of cryptotanshinone and reduce the bioavailability of cryptotanshinone (Wang et al., 2023).

## 4 Anti-inflammatory activities

In colitis mouse model, OP-D ameliorates the colitis by inhibiting epithelial NF-κB signaling pathway (Wang et al., 2022). In streptozotocin-induced diabetic nephropathy rats, OP-D ameliorates renal function by inhibiting inflammatory response (Qiao et al., 2020). Related studies have shown that particulate matter with a diameter of less than 2.5 µm (PM2.5) can cause lung inflammation. In mouse pulmonary epithelial cells, OP-D significantly ameliorates PM2.5-induced inflammation by inhibiting the AMPK/NF-κB signaling pathway (Wang Y. et al., 2020). Atopic dermatitis (AD) is a prevalent condition globally, marked by symptoms such as itching and eczema. In atopic dermatitis mouse models and inflamed HaCaT cells, OP-D can treat inflammatory skin diseasessuch as AD (An et al., 2020). In addition, related studies have observed the anti-inflammatory effects of OP-D in the spleen. The level of cytokine expression in the blood of OP-D-treated mice is significantly decreased (An et al., 2020).

## 5 Anti-cancer activities

**FIGURE 3:**
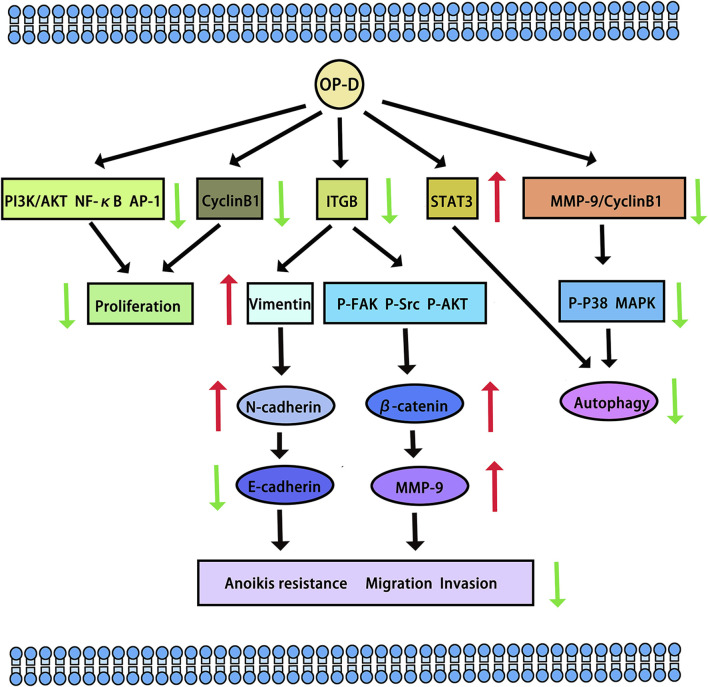
The potential anti-cancer effects and mechanisms of OP-D. The red arrow means up-regulation, the green arrow means down-regulation.

Tumours pose a significant threat to human health. In a study by Zhang Y et al., the potential of OP-D to inhibit melanoma was explored. The research revealed that OP-D effectively suppressed both the invasion and proliferation of MDA-MB-435 melanoma cells. Additionally, OP-D was found to inhibit the adhesion of these cells to fibronectin. Mechanically, OP-D suppressed the phosphorylation of p38 and the expression of matrix metalloproteinase-9 (MMP-9) (Zhang Y. et al., 2015). Exploring the anti-metastasis effect of OP-D in triple-negative breast cancer (TNBC) cells and its mechanism should be endowed with paramount importance. OP-D inhibited the migration, invasion and proliferation of MDA-MB-231 cells. Mechanically, OP-D upregulated the nuclear β-catenin and reduced the phosphorylation of FAK/Src/AKT by abolishing ITGB1 expression (Zhu et al., 2020). In addition, the number of viable cells and colony formation significantly decreased when the cells were exposed to OP-D. OP-D inhibits the proliferation of MCF-7 cells and causes cell cycle arrest at the G (2)/M phase. Mechanically, cyclin B1 downregulation was linked to OP-D-induced G (2)/M cell cycle arrest. Moreover, OP-D-induced apoptosis included the activation of caspase-8 and caspase-9 (Zang et al., 2016). OP-D may be a promising natural anti-cancer agent for the treatment of colorectal cancer and throat cancer. In human laryngocarcinoma cells, OP-D boosted caspase-3/9 activity, induced apoptosis, promoted cytotoxicity, and decreased cell growth. OP-D markedly elevated p-p38 MAPK protein expression while dramatically downregulating the expression of cyclin B1 and matrix metalloproteinase-9 (MMP-9) proteins (Yan et al., 2019). Ko HM et al. aimed to investigate the anti-colorectal cancer effect of OP-D. They found that OP-D (20–40 uM) significantly inhibits cell viability and possesses anti-proliferative properties. By preventing IPO7 and XPO1 from being produced, OP-D (40 uM) caused nucleolar stress and suppressed the expression of Ki67 (a marker for cell proliferation). Furthermore, OP-D controlled CDK4 and cyclin D1. Furthermore, in a dose-dependent manner, OP-D consistently suppressed the phosphorylation of AKT expression. The T_1/2_ of c-Myc was shortened by OP-D in a time-dependent way (Ko et al., 2022). In human lung cancer cells, OP-D suppresses the proliferation of cells and reduces the expression of several carcinogenic gene products by inhibiting the NF-κB, PI3K/AKT, and AP-1 pathways (Lee et al., 2018b). Another study suggests that OP-D can induce apoptosis and exert anti-tumor effects by inhibiting of signal transducer and activator of transcription 3 (STAT3) signaling pathways in non-small cell lung carcinoma (NSCLC) cells (Lee et al., 2018a) (The potential anti-cancer effects and mechanisms of OP-D are shown in Figure 3).

## 6 Cardiovascular protection activities

OP-D is recognized as the principal bioactive constituent of traditional Chinese medicine formulations such as Shenmai san, Shenmai injection (SM-I), and Radix *Ophiopogon japonicus* (Jiang et al., 2014). The study demonstrated that OP-D attenuated doxorubicin-induced cardiomyocyte injury by suppressing endoplasmic reticulum stress (ERS) and relieving mitochondrial damage (Meng et al., 2014). Mechanically, OP-D mitigated autophagy activity by diminishing the production of reactive oxygen species (ROS) (Zhang YY. et al., 2015). Another study pointed out that OP-D reduced diabetic myocardial injuries by regulating the dynamics of mitochondria. In type 2 diabetes mice, OP-D lowered blood lipid levels and alleviated mitochondrial dysfunction. In myocardial lipotoxicity models, OP-D inhibited the mitochondrial dysfunction and promoted the cell survival rate (Li et al., 2021). Furthermore, OP-D also prevented H_2_O_2_-induced injury in human umbilical vein endothelial cells (HUVECs), where OP-D dose-dependently reduced the mRNA levels of anti-oxidant, pro-inflammatory, and apoptotic genes. Pretreatment with OP-D reduced H_2_O_2_-induced lipid peroxidation and protein carbonylation. In addition, in cells treated with OP-D, mitochondrial ROS production and cell death were diminished. Furthermore, OP-D prevented the release of inflammatory cytokines and restored the entire antioxidative capacity of the cell (Qian et al., 2010). Zhang GC found that OP-D protected isoproterenol-induced cardiomyocyte injury by regulating multiple signaling pathways of target proteins (Zhang et al., 2022). Ophiopogon-D′ (OP-D′) and OP-D are the two main active components in *Ophiopogon japonicus*, and these factors have the same molecular formula and similar structures. Interestingly, OP-D′ induced cardiomyocyte mitophagy and mitochondrial damage (Lei et al., 2022). OP-D protected against OP-D′-induced cardiomyocyte injury through the inhibition of ERS. The rate of apoptosis was significantly increased by OP-D′ (6 uM) and genes related to ERS had increased expression. The endoplasmic reticulum’s morphology was altered, and myocardial cell damage caused by OP-D′ could be partially mitigated at varying concentrations of OP-D (Wang et al., 2019).

**FIGURE 4:**
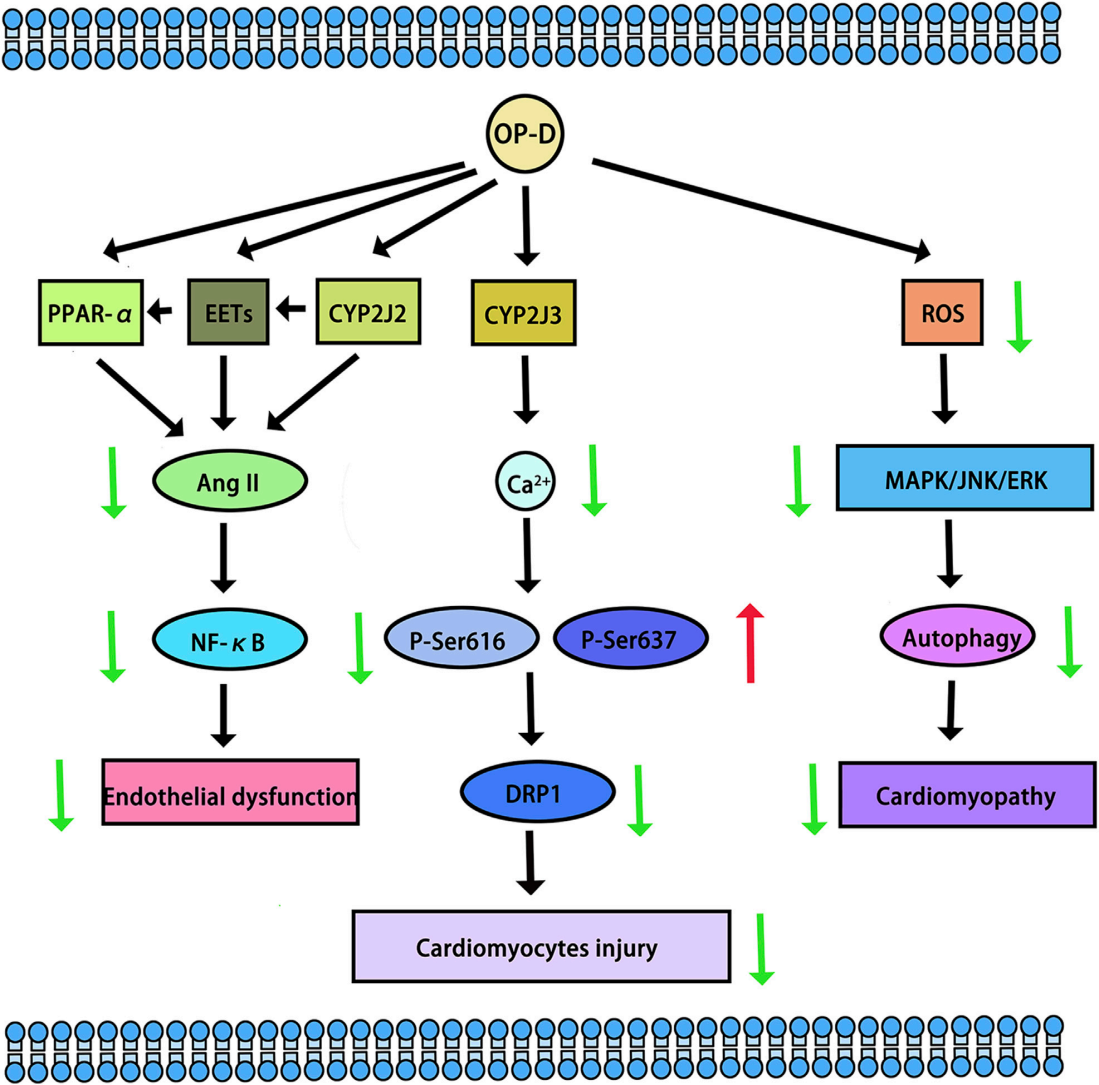
The cardiovascular protective mechanisms of OP-D. The red arrow means up-regulation, the green arrow means down-regulation.

CYP2J2, CYP4F3, CYP4A11, CYP4A22, CYP4F2, and CYP4F3 were common fatty acid metabolic enzymes in cardiomyocytes. The research revealed that low concentrations of OP-D did not impact the viability of cardiomyocytes. Conversely, concentrations of OP-D exceeding 20 µM could potentially enhance cell viability. At concentrations below 100 μM, OP-D did not significantly alter the morphology or quantity of cardiomyocytes. At 5 μM, OP-D mildly upregulated CYP2J2 and CYP4F3 mRNA expression, whereas high concentrations of OP-D substantially enhanced these expressions in a dose-dependent manner. On the mRNA expressions of CYP4A11, CYP4A22, and CYP4F2, the same concentration of OP-D had a minor impact. In a dose-dependent manner, 20 uM OP-D might considerably increase the expression of CYP4F3 in the protein (Tang et al., 2021). CYP2J2 was highly expressed in cardiovascular tissue. Huang X et al. found that OP-D has an endothelial protective effect via activating the CYP2J2-PPARα pathway in HUVECs. By upregulating CYP2J2/EETs and PPARα in HUVECs, OP-D dramatically reduced Ang II-induced NF-κB nuclear translocation, IκBα downregulation, and activation of pro-inflammatory cytokines (TNF-α, IL-6, and VCAM-1) (Huang et al., 2017). Another study demonstrated OP-D’s ability to inhibit Angiotensin II (Ang II)-induced vascular endothelial cell death by upregulating CYP2J2 (Huang XY. et al., 2018). Meanwhile, OP-D was closely related to CYP2J3. By inhibiting inflammation *in vivo* and upregulating CYP2J3 *in vitro*, OP-D reduced ventricular hypertrophy in rats. Ang II treatment was administered to H9c2 cells. In response to Ang II-induced hypertrophy, specific hypertrophy genes and NF-κB signaling molecules were expressed at higher levels. Nevertheless, OP-D combined with Ang II negated these inductive effects (Wang et al., 2018). By upregulating CYP2J3, OP-D also reduced Myocardial Ischemia-Reperfusion (MI/R) Injury in rats. OP-D provided a range of preventive measures against MI/R injury. These included improving the healing of damaged myocardial structures, reducing the synthesis of creatine kinase and lactate dehydrogenase, attenuating the size of myocardial infarcts, and regulating heart function. There was potential in developing OP-D as a unique medication for the treatment of MI/R damage (Huang X. et al., 2018). Another study demonstrated that OP-D upregulated CYP2J3 and suppressed ER stress in rat cardiomyocytes to preserve Ca^2+^ homeostasis *in vitro* (You et al., 2016). Besides, Wang, J et al. found that OP-D could increase SERCA2a interaction with phospholamban by inducing the increase of CYP2J3 in rat cardiomyocytes. Through increasing SERCA2a activity and phosphorylating PLB, the occurrence of heart failure was reduced. In a heart failure model, the reduction of CYP2J3 eliminated these positive effects of OP-D on heart failure (Wang J. et al., 2020) (The cardiovascular protective mechanisms of OP-D are shown in Figure 4).

## 7 Bone protection activities

OP-D is also a new herbal agent against osteoporosis. The research showed that OP-D markedly increased the proliferation of MC3T3-E1 cells. Furthermore, in RAW264.7 cells, both TRAP activity and the mRNA expressions of osteoclastic genes were decreased. One of the main factors contributing to the development of osteoporosis was an excess of ROS. In MC3T3-E1 cells and RAW264.7 cells, OP-D inhibited the generation of ROS. Serum bone degradation indicators, such as TRAP and CTX-1, showed decreased activity following OP-D treatment. Subsequent investigations revealed that OP-D exhibited anti-osteoporosis properties by lowering ROS via the FoxO3a-β-catenin signaling pathway (Huang et al., 2015). Clinical evidence has indicated a high failure rate of titanium implants in diabetic patients. Under diabetic conditions, excessive oxidative stress at the bone-implant interface plays an important role in the impaired osteointegration. OP-D ameliorated the osteointegration of titanium alloy implants by preventing ROS overproduction through the Wnt/β-catenin signaling pathway (Ma et al., 2018). PECAM1 (platelet and endothelial cell adhesion molecule 1) holds considerable significance for angiogenesis and osteogenesis and is involved in bone regeneration. Endothelial-specific KLF3 knockout mice showed increased PECAM1 and accelerated bone formation in the bone regeneration area. As a KLF3 inhibitor, OP-D stimulated the formation of vessels both *in vivo* and *in vitro*. When OP-D was administered, PECAM1 abundance rose and bone healing accelerated. These findings offered a novel therapeutic target for the treatment of bone fractures and the enhancement of bone regeneration (Yang et al., 2020).

## 8 Other activities

Atherosclerosis is a common cardiovascular disease. OP-D plays an important role in atherosclerosis. When compared to the model group, OP-D dramatically reduced the amount of serum lipid and the formation of plaque. Furthermore, OP-D decreased hepatocyte steatosis while enhancing insulin resistance and oral glucose tolerance. Subsequent investigation showed that OP-D might reduce atherosclerosis by blocking mTOR phosphorylation and targeting lipid metabolism pathways regulated by SREBP1 and SCD1, as observed in both vivo and vitro. The gut microbiota was confirmed to be crucial in atherosclerosis pathogenesis. In HFD-fed mice, OP-D treatment resulted in notable structural alterations in the gut microbiota and faecal metabolites. Additionally, it decreased the relative abundance of Erysipelotrichaceae genera linked to the metabolism of cholesterol (Zhang et al., 2021).

Gut dysbiosis also plays a critical role in the pathogenesis of obesity. In HFD-fed mice, OP-D ameliorated body weight, hyperglycemia, hyperlipidemia, and insulin resistance. Specifically, OP-D reversed HFD-induced gut dysbiosis (Chen et al., 2018).

Nonalcoholic fatty liver disease (NAFLD), a clinicopathologic syndrome characterized by excessive fat deposition in the liver cells, not caused by alcohol or other liver-specific toxins. In HFD-fed obese mice, OP-D reduced NAFLD by enhancing oxidative stress, lipid metabolism, and inflammatory response. *In vitro*, OP-D treatment lowered the levels of inflammation and lipogenesis. One possible explanation for OP-D’s beneficial effects on NAFLD was the NF-κB signaling pathway (Huang et al., 2023).

Systemic lupus erythematosus (SLE) is an autoimmune disease. The activation of autoreactive B cell differentiation will promote the development of SLE. In MRL/lpr mice, the treatment of OP-D decreased the serum levels of IgG, IgM, and anti-dsDNA autoantibodies. Meanwhile, OP-D improved the progression of SLE by decreasing the number of B cells (Nie et al., 2023).

OP-D is a potential anti-pulmonary fibrosis drug. *In vivo* and *in vitro* models, OP-D inhibited epithelial-mesenchymal transition and excessive extracellular matrix deposition, accelerated lung fibroblast apoptosis, and prevented lung fibroblasts from differentiating into myofibroblasts. According to multi-omics techniques and bioinformatics analysis, the AKT/GSK3β pathway was inhibited by OP-D. OP-D combined with PI3K/AKT inhibitors could effectively alleviate pulmonary fibrosis (Bao et al., 2023).

OP-D can reduce the excitability of airway parasympathetic ganglion neurons. The hyperpolarizing effect of OP-D on paratracheal neurones by activating the potassium conductance might explain the mechanisms of the antitussive effect (Ishibashi et al., 2001). By directly affecting airway epithelial cells, OP-D can also enhance mucin secretion and production (Park et al., 2014).

## 9 Discussion

As mentioned above, OP-D has a wide range of pharmacological effects, including anti-cancer, anti-inflammatory, bone protection and cardiovascular protection. OP-D has a potential value in the prevention and treatment of many diseases (Table 1). In addition, OP-D also plays an important role in other aspects. Ginsenosides Rb1, Ginsenosides Rd, rosuvastatin, and glycyrrhizic acid significantly reduce the uptake of OP-D in liver (Zhang et al., 2017; Liu et al., 2018). As an ingredient of vaccines, adjuvants can directly stimulate or promote the immune responses. It has been discovered that OP-D works well as a vaccine adjuvant. The problems of the low solubility and toxicity of OP-D can be effectively overcome by using a low-energy emulsification method to prepare nanoemulsion OP-D (Tong et al., 2018; Luo et al., 2022). Interestingly, OP-D and OP-D′ act as isomers of each other. *In vitro* studies showed that only OP-D′ induced a hemolysis reaction, whereas *in vivo*, both OP-D and OP-D′ were found to cause hemolysis. The hemolytic effects of OP-D and OP-D′ were thought to be closely associated with disruptions in phospholipid metabolism (Xu et al., 2021). In some difficult problems, such as the osteointegration of titanium alloy implants, the studies have also confirmed the application value of OP-D. Nevertheless, there are still many questions of OP-D that need to be discussed. On the one hand, the toxicity testing of OP-D in animals is currently insufficient. Yu J et al. assess the long-term toxicokinetic profiles of the OP-D in SM-I. They found that OP-D exhibited an extremely low exposure level and a rapid elimination rate after injection (Yu et al., 2014). As the main component of *Ophiopogon japonicus*, we speculate that OP-D may have side effects such as gastrointestinal reactions and allergic reactions like *Ophiopogon japonicus*. On the other hand, the anti-cancer effects and mechanisms of OP-D require further investigation. Future research is anticipated to ensure OP-D’s safety and efficacy in protecting human health.

**TABLE 1:** Pharmacological activities of OP-D.

Subjects	Pharmacologic action	Function	Ref.
Male C57BL/6 J mice IEC-6 cells	Anti-colitis	GSH ↑ SOD ↑ TNF-α ↓ IL-1β ↓ IL-6 ↓	Wang et al. (2022)
Male Sprague Dawley rats	Anti-diabetic nephropathy	GSH ↑ SOD ↑ CAT ↑ TNF-α ↓ TNF-α ↓	Qiao et al. (2020)
MLE-12 cells	Anti-lung inflammation	TNF-α ↓ IL-1β ↓ IL-6 ↓ IL-8 ↓	Wang J. et al. (2020)
Female BALB/c mice HaCaT cells	Anti-atopic dermatitis	TNF-α ↓ IL-1β ↓ IL-6 ↓	An et al. (2020)
MDA-MB-435 cells	Anti-breast cancer	Cells proliferation ↓ invasion ↓ migration ↓	Zhang et al. (2015a)
MDA-MB-231 cells	Anti-breast cancer	Cells proliferation ↓ invasion ↓ migration ↓	Zhu et al. (2020)
MCF-7 cells	Anti-breast cancer	apoptosis ↑	Zang et al. (2016)
AMC-HN-8 cells	Anti-laryngocarcinoma	Cells proliferation ↓ apoptosis ↑	Yan et al. (2019)
HCT116 cells	Anti-colorectal cancer	Cells proliferation ↓ apoptosis ↑	Ko et al. (2022)
H1299 cells A549 cells	Anti-lung cancer	Cells proliferation ↓ apoptosis ↑	Lee et al. (2018a)
H1299 cells A549 cells H460 cells	Anti-lung cancer	Cells proliferation ↓ apoptosis ↑	Lee et al. (2018b)
H9C2 cells	Cardiovascular protection	ROS ↓ ATF6α ↓ GRP78 ↓ CHOP ↓	Meng et al. (2014)
C57BL/6 J mice H9C2 cells	Cardiovascular protection	Autophagy ↓ ROS ↓ EF ↑ LVFS ↑	Zhang et al. (2015b)
Male db/db mice Male C57BL/6 J mice H9C2 cells	Cardiovascular protection	ALT ↓ MFN1 ↑ MFN2 ↑ OPA1 ↑	Li et al. (2021)
Human umbilical vein endothelial cells	Cardiovascular protection	ROS ↓ HO-1 ↓ PGC-1α ↓	Qian et al. (2010)
Human umbilical vein endothelial cells	Cardiovascular protection	TNF-α ↓ IL-6 ↓ VCAM-1 ↓	Huang et al. (2017)
Male Sprague-Dawley rats H9C2 cells	Cardiovascular protection	ANP ↓ BNP ↓ β-MHC ↓	Wang et al. (2018)
Male Sprague-Dawley rats	Cardiovascular protection	LDH ↓ SOD2 ↓ CK ↓	Huang X. et al. (2018)
H9C2 cells	Cardiovascular protection	SERCA2a ↑ PLB ↑ RyR2 ↑ FKBP12.6 ↑	You et al. (2016)
H9C2 cells	Cardiovascular protection	SERCA2a ↑ PLB ↑	Wang Y. et al. (2020)
Female BALB/c mice MC3T3-E1 cells RAW264.7 cells	Bone protection	ROS ↓ Calciumdeposition ↑ ALP ↑ NFATc1 ↑ TRAP ↑ CTX-1 ↑	Huang et al. (2015)
Male New Zealand rabbits Rabbit osteoblasts	Bone protection	ALP ↑ Runx2 ↑ Osterix ↑ Col 1 ↑ OPN ↑ SOD ↓ ROS ↓	Ma et al. (2018)
Cdh5-Cre transgenic mice Human microvascular endothelial cells	Bone protection	CD31 ↑ EMCN ↑ JUNB ↑ VEGFA ↑ VEGFB ↑ PDGFA ↑ PDGFB ↑	Yang et al. (2020)
Male ApoE^−/−^ mice Male C57BL/6 mice Human LO2 cells	Anti-atherosclerosis	TG ↓ TC ↓ LDL-C ↓ MDA ↓ LDH ↓ ALT ↓ AST ↓	Zhang et al. (2021)
Male C57BL/6 J mice	Anti-obesity	TG ↓ TC ↓ LDL-C ↓ TNF-a ↓ MCP-1 ↓ ALT ↓ AST ↓	Chen et al. (2018)
Male C57BL/6 J mice Primary hepatocytes	Anti-NAFLD	TG ↓ TC ↓ FFA ↓ LDL-C ↓ SOD ↓ TNF-α ↓ IL-1β ↓ IL-6 ↓	Huang et al. (2023)
Female MRL/lpr mice	Anti-systemic lupus erythematosus	Proteinuria levels ↓ Serum creatinine levels ↓	Nie et al. (2023)
Male C57BL/6 J mice	Anti-pulmonary fibrosis	GSH-PX ↑ MDA ↓ HIF-1α ↓ IL-6 ↓ TNF-α ↓ α-SMA ↓	Bao et al. (2023)
Wistar rats	Antitussive effect	Excitability of airway parasympathetic ganglion neurons ↓	Ishibashi et al. (2001)
NCI-H292 cells	Antitussive effect	Mucin production and secretion ↑	Park et al. (2014)

## Abbreviations

AD, Atopic dermatitis; Ang II, Angiotensin II; DMSO, Dimethyl sulfoxide; DT-13, Dwarf lilyturf tuber-13; ERS, Endoplasmic reticulum stress; ESI-MS, Electrospray ionization mass spectrometry; HUVECs, Human umbilical vein endothelial cells; LC, Liquid chromatography; LP-B, Liriopesides B; MCF-7, Human breast carcinoma-7; MI/R, Myocardial Ischemia-Reperfusion; MMP-9, Matrix metalloproteinase; NSCLC, Non-small cell lung carcinoma; NAFLD, Nonalcoholic fatty liver disease; OATPs, Organic anion-transporting polypeptides; OP-B, Ophiopogon-B; OP-D, Ophiopogon-D; RUS, Ruscogenin; OP-D′, Ophiopogon-D′; PECAM1, Platelet and endothelial cell adhesion molecule 1; ROS, Reactive oxygen species; STAT3, Signal transducer and activator of transcription 3; SM-I, Shenmai injection; SLE, Systemic lupus erythematosus; TNBC, Triple-negative breast cancer.
